# Serum Cytokeratin 19 Fragment, CK19-2G2, as a Newly Identified Biomarker for Lung Cancer

**DOI:** 10.1371/journal.pone.0101979

**Published:** 2014-07-09

**Authors:** Jia Gao, Fang Lv, Jia Li, Zongyong Wu, Jun Qi

**Affiliations:** 1 Clinical Laboratory, Cancer Institute and Hospital, Chinese Academy of Medical Sciences and Peking Union Medical College, Beijing, China; 2 Department of Thoracic Surgery, Cancer Institute and Hospital, Chinese Academy of Medical Sciences and Peking Union Medical College, Beijing, China; Ludwig-Maximilians University, Germany

## Abstract

**Background:**

CK19-2G2, a new fragment of cytokeratin 19, is a potential tumor marker for diagnosing lung cancer. The preoperative level of serum CK19-2G2 has been demonstrated to be associated with tumor metastasis and survival of breast cancer patients. This study investigated the postoperative dynamic changes in serum CK19-2G2 levels and its clinical significance in lung cancer patients.

**Materials and Methods:**

Preoperative serum CK19-2G2 levels were measured in 630 lung cancer patients and were compared with individuals with benign pulmonary diseases (n = 134) and healthy volunteers (n = 263). In 352 cases, the patients underwent surgery. In these patients, in addition to preoperative assays, serum CK19-2G2 was also monitored at 1 week and 1 month after the operation.

**Results:**

The preoperative baseline levels of serum CK19-2G2 was significantly higher in lung cancer patients than patients with benign diseases and healthy controls (P<0.001). The postoperative levels of CK19-2G2 declined significantly within 1 week after tumor resection. Hereafter, a further decrease was observed in the patients who underwent palliative operations, while for the patients in the radical resection group, their CK19-2G2 levels stabilized.

**Conclusion:**

CK19-2G2 may be a candidate marker for diagnosing and monitoring a patient's response to lung cancer treatment. In addition, CK19-2G2 may be an indicator for micrometastases in lung cancer patients.

## Introduction

Lung cancer is the second most common cancer in the United States, with an incidence of 84.4/100,000 men and 55.7/100,000 women, and represents the leading cause of death for cancer [1]. It is also one of the main causes of cancer mortality and incidence in China. Surgery, chemotherapy and radiotherapy are the main therapeutic methods used for treating patients with lung cancer [2]. During treatment, medical imaging can be used to directly reflect the size and morphological changes of the tumor; however, it is meaningless for analyzing tumor cell activity. Some biomarkers, such as cytokeratins, can reflect ongoing cell activity and are therefore useful for monitoring treatment.

Cytokeratins belong to the family of intermediate filament (IF) proteins [3]. At present, more than 20 different cytokeratins have been identified, of which cytokeratin 19 has been shown to be the most abundant in simple epithelial cells [4]. An assay measuring soluble cytokeratin 19 fragments in the circulation, CYFRA 21-1, is a particularly useful tool in lung cancer [5], [6] and is mainly used in monitoring treatment and evaluating response to therapy in lung cancer. Recently, a new fragment of cytokeratin 19, named CK19-2G2, was identified by two monoclonal antibodies. In our previous study, we assessed the serum concentration of CK19-2G2 and Cyfra21-1 in 104 lung cancer patients, 71 patients with benign lung diseases and 105 healthy controls. We found that CK19-2G2 was elevated in lung cancer patients and was superior to Cyfra21-1 in diagnosing squamous cell carcinoma [7]. The purpose of the present study is to assess the clinical value of CK19-2G2 in monitoring treatment effects after surgery. Therefore, we compared the preoperative and postoperative concentrations of CK19-2G2 in lung cancer patients who had undergone a pulmonary resection.

## Materials and Methods

### Patient cohort

Table 1 shows the tested groups. Serum specimens were obtained from lung neoplasm patients and who were diagnosed pathologically between February 2011 and March 2013 at the Department of Thoracic Surgery, Cancer Institute and Hospital, Chinese Academy of Medical Sciences and Peking Union Medical College (CAMS, Beijing, China). Patients who had received preoperative radiotherapy or chemotherapy were excluded. The histological classification was made according to the WHO/IASLC classification criteria for lung cancer. Prior to surgery, peripheral blood was collected from 630 lung cancer patients (T0), including 345 with adenocarcinoma, 208 with squamous cell carcinoma, 36 with small cell lung cancer (SCLC), and 41 with other histological types. The median age was 59.37 years (range, 20–83 years). According to the seventh edition of the Tumor-Node-Metastasis (TNM) staging classification for lung cancer, 226 patients were in stage I, 126 were in stage II, 145 were in stage III, and 18 were in stage IV (Table 1).

**Table 1:** Demographics and clinical characteristics of study population.

Characteristics	Lung cancer	Benign diseases	Healthy controls
Total	630	134	263
Age (year)			
Mean	59.37	58.24	52.12
Range	20–83	30–81	22–65
Sex			
Male	425	96	179
Female	205	38	84
Lung cancer stage			
Stage I	226	NA	NA
Stage II	126	NA	NA
Stage III	145	NA	NA
Stage IV	18	NA	NA
Missing	115	NA	NA
Histology			
Malignant			
Adenocarcinoma	345	NA	NA
Squamous carcinoma	208	NA	NA
SCLC	36	NA	NA
Others	41	NA	NA
Benign			
Pneumonia	NA	43	NA
Pulmonary tuberculosis	NA	66	NA
Benign tumor	NA	25	NA

The 352 patients underwent one of the following surgical procedures: lobectomy with lymph node dissection (radical resection, with pathologically confirmation that no residual tumor remained, n = 321), partial resection of the lung without lymph nodes dissection (palliative operation, n = 31, including 21 with visible residual tumor mass, and 10 with residual metastatic lymph nodes). On postoperative day 7 (T1), peripheral blood was collected from all patients. In some of these patients (n = 101), blood was also collected one month after surgical resection (T2), during scheduled medical examinations.

The control groups were comprised of 134 patients with benign pulmonary diseases and 263 healthy volunteers. The histopathology of the benign pulmonary diseases was established by tissue biopsy or after surgery. There were 43 patients with pneumonia, 66 with pulmonary tuberculosis, and 25 with pulmonary benign tumors. The median age of the patients with benign pulmonary diseases was 58.24 years (range, 30–81 years). Peripheral blood was collected before treatment was performed.

The healthy control subjects were recruited from individuals who visited the department of cancer prevention of CAMS for a routine health check-up between February 2011 and March 2012. The median age was 52.12 years (range, 22–65). Our study was approved by the Chinese Academy of Medical Sciences Cancer Hospital Ethnic Committee. All participants provide their written informed consent to participate in this study and for the case details to be published.

### Sample collection

Venous blood (5 ml) was obtained from each patient and placed in test tubes without anticoagulants. Within 2 h of sample collection, the serum was separated from each blood sample by centrifuging at 4000 rpm for 10 min. All specimens were stored at −70°C until analysis.

### Determination of serum CK19-2G2

The serum CK19-2G2 concentration was measured using the Chemical luminescence immunity analyzer (Tongsheng Times, Peking, China) and the CK19-2G2 Chemiluminescence Quantitative Immunoassay (CLIA) kit (Tongsheng Times, Peking, China). The kit was comprised of two monoclonal antibodies, CK19-2G2 (CK19 aa 375–400) and CK19-5H2 (CK19 aa 325–350). The detection protocol was carried out according to the manufacturer's instructions and in duplicate for calibrators and controls. The appropriate controls were within the ranges provided by the manufacturer.

### Statistical analysis

The statistical analysis was performed using the SPSS software, version 11.0. The data are presented as the mean±standard deviation (SD). Nonparametric methods were used to compare the levels of CK19-2G2 between the different groups of patients. Comparisons between two groups were performed using the Mann-Whitney test. In case of multiple groups, Kruskal-Wallis tests were used, with post hoc comparisons according to Mann-Whitney method. Receiver operating characteristic (ROC) curves were constructed to estimate the diagnostic performance of CK19-2G2 in discriminating lung cancer patients from healthy individuals and patients with benign pulmonary diseases. The CK19-2G2 concentrations for each patient at different time points were compared with using the paired *t*-test. Statistically significant differences were defined as comparisons resulting in *P*<0.05.

## Results

### Patient characteristics

The demographics and clinical characteristics of the study groups are shown in Table 1. There were no significant differences in age and sex ratio among the three groups.

### Baseline Preoperative Serum CK19-2G2 levels

**Figure 1:**
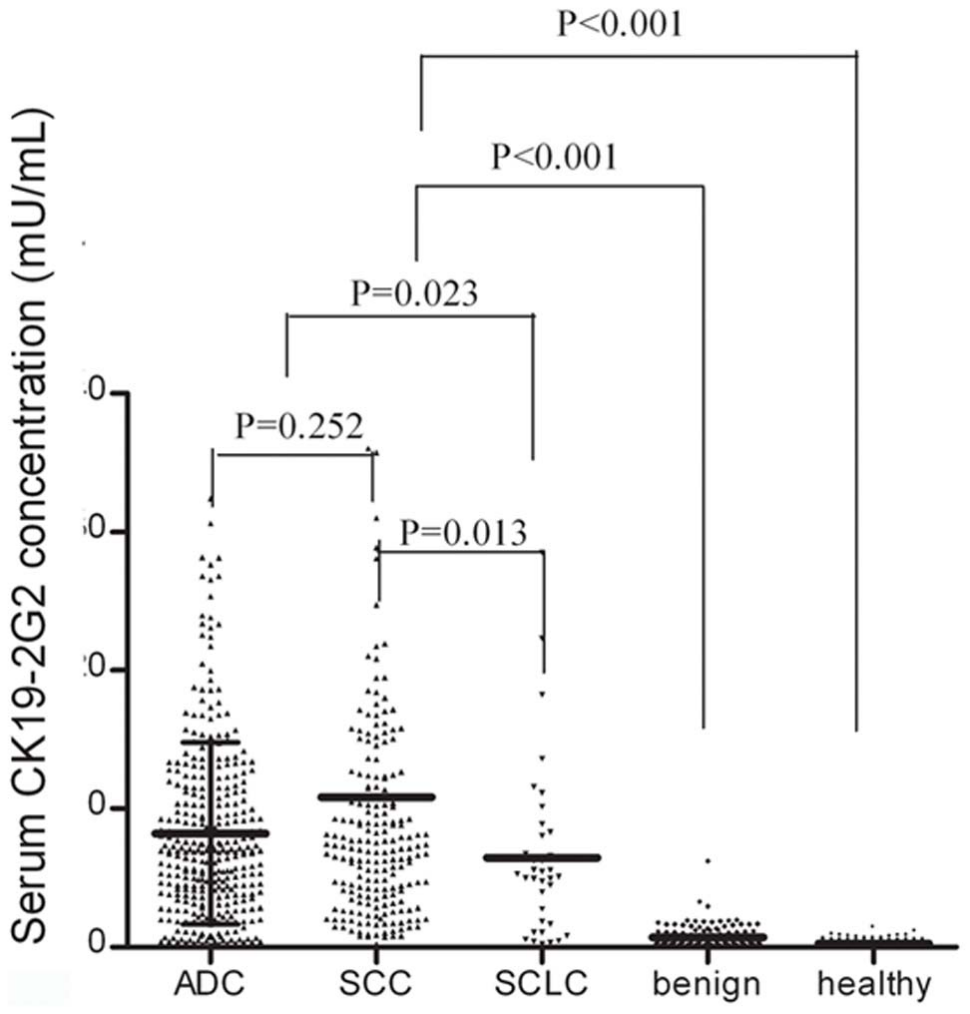
Serum CK19-2G2 concentrations in lung cancer patients, patients with benign diseases and healthy controls. The scatter represents a single value and the lines represent the mean levels of CK19-2G2 for the benign, healthy and malignant histology subtypes. Abbreviations: ADC: adenocarcinoma; SCC: squamous carcinoma; SCLC: small cell lung cancer.

The comparison of serum CK19-2G2 concentrations in each studied groups is shown in Fig. 1. The baseline preoperative serum CK19-2G2 level in lung cancer patients was significantly higher than that of benign disease patients (8.92±9.95 vs. 0.71±0.78 mU/mL; *P*<0.001) and that of the healthy control group (8.92±9.95 vs. 0.26±0.19 mU/mL; *P*<0.001) (Table 2, and the full data is shown in Table S1). When using benign disease patients and healthy controls as the comparison group for all cases, the AUC value for the ROC curve was 0.971 (95% CI: 0.962–0.980).

**Table 2:** The mean level of CK19-2G2 for lung cancer, benign diseases and healthy controls.

Group	N	CK19-2G2	
		Mean (mU/mL)	SD
Case (pre-operation)	630	8.92	9.94
NSCLC	553	9.20	10.27
ADC	345	8.21	6.57
SCC	208	10.83	14.30
SCLC	36	6.46	6.23
Benign disease	134	0.71	0.78
Healthy	263	0.26	0.19

Abbreviation: NSCLC = non-small cell lung cancer; ADC = adenocarcinoma; SCC = squamous cell carcinoma; SCLC = small cell lung cancer.

Among the different histological types of lung cancer, patients with non-small cell lung cancer (NSCLC), which is composed of adenocarcinoma and squamous cell carcinoma, had a higher CK19-2G2 level than patients with SCLC (9.20±10.27 vs. 6.46±6.23 mU/mL, *P* = 0.023) (Table 2). The difference between the CK19-2G2 serum levels of the adenocarcinoma and squamous cell carcinoma patients was not statistically significant (8.21±6.57 vs. 10.83±14.30, *P* = 0.252).

### Dynamic Changes of Serum CK19-2G2 Levels after Tumor Resection

The preoperative and postoperative serum levels of CK19-2G2 for each group are summarized in Table 3 (The full data is shown in Table S2). In total, 352 patients underwent surgical procedures, and the patient's peripheral blood was collected 1–2 days before surgery (T0) and 1 week after surgery (T1). The mean CK19-2G2 concentration was significantly higher at T0 than at T1 (10.24±8.68 vs. 0.62±1.94, P<0.001).

**Table 3:** Serum CK19-2G2 concentration in the lung cancer group before and after operation.

Group	N	CK19-2G2	
		Mean (mU/mL)	SD
Pre-operation (T0)	352	10.24	8.68
radical resection	321	10.19	8.43
palliative operation	31	10.70	11.07
1 week after operation (T1)	352	0.62	1.94
radical resection	321	0.47	0.55
palliative operation	31	2.21	6.16
1 month after operation (T2)	101	0.49	0.61
radical resection	89	0.43	0.43
palliative operation	12	0.99	1.24

Note: T0 vs. T1 & T2: P<0.001; radical resection (T0) vs. palliative operation (T0): *P* = 0.617; radical resection (T1) vs. palliative operation (T1): *P*<0.001; radical resection (T2) vs. palliative operation (T2): *P* = 0.090.

**Figure 2:**
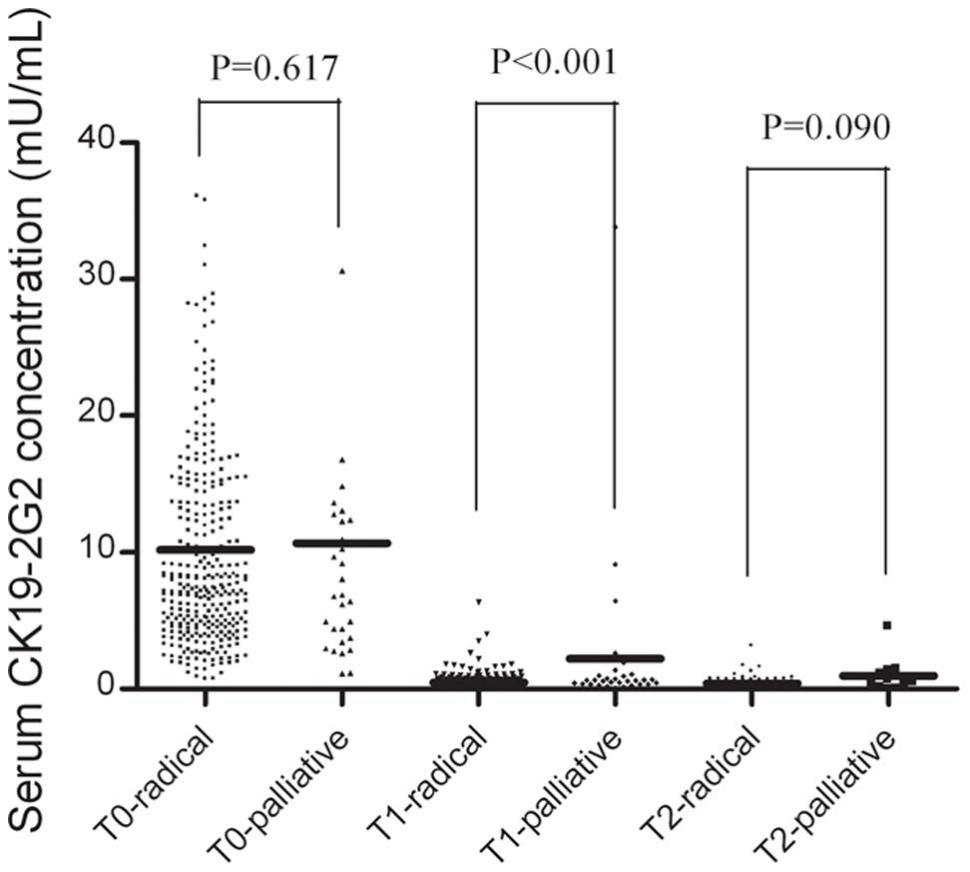
Dynamic changes in serum CK19-2G2 levels in lung cancer patients before and after surgery. The scatter represents a single value and the lines represent the mean levels of CK19-2G2 in lung cancer patients who received radical or palliative operations.

In addition, subgroup analyses based on surgery procedures (radical resection vs. palliative operation) were performed to study the possible impact of operation mode on postoperative CK19-2G2 levels. In the T0 group, the mean preoperative level of CK19-2G2 (mU/mL) was 10.19±0.47 in radical resection subgroup, and 10.70±1.99 in palliative operation subgroup. No significant difference was found between the two subgroups (P>0.05) (Fig. 2). In the T1 group, the serum CK19-2G2 (mU/mL) level was significantly lower in the radical resection subgroup, compared with that in palliative operation subgroup (0.47±0.55 vs. 2.21±6.16, *P*<0.001) (Fig. 2). For each patient, the postoperative CK19-2G2 level was significantly lower than that before surgery, regardless of the surgery procedure chosen (*P*<0.001, paired *t*-test).

For 101 patients, blood was collected three times in the clinical process, specifically, preoperatively (T0) and 1 week (T1) and 1 month after surgical resection (T2). Of the study cohort, the mean CK19-2G2 concentration of the T1 and T2 groups was significantly lower than that of the T0 group (T1: 0.82±3.42 & T2: 0.49±0.61 vs. T0: 8.36±8.31, *P*<0.001, Data was not shown in Tables). No significant difference was observed between the T1 and T2 groups (*P* = 0.236). In the T2 group, the serum CK19-2G2 (mU/mL) level was lower in the radical resection subgroup, compared to the palliative operation subgroup (0.43±0.43 vs. 0.99±1.24, *P* = 0.090, Table 3). In the palliative operation subgroup, the CK19-2G2 level of the T2 group was significantly lower than that in the T1 group (0.99±1.24 vs. 2.21±6.16, *P*<0.01, paired *t*-test). No similar results were observed in the radical resection subgroup (T1: 0.47±0.55 vs. T2: 0.43±0.43, *P*>0.05).

## Discussion

In the present study, we investigated the role of CK19-2G2 in diagnosing and monitoring the surgical effects of lung cancer. Our results are summarized as follows: Firstly, the baseline preoperative serum CK19-2G2 of lung cancer patients was significantly higher than that of patients with benign diseases and healthy controls. Secondly, the postoperative CK19-2G2 levels declined significantly within 1 week after tumor resection, no matter which surgery procedure was performed. Accordingly, further decreases were observed for the patients who underwent palliative operation, while for the radical resection group, the CK19-2G2 level stabilized.

When we compared the preoperative and postoperative concentrations of CK19-2G2, we found that postoperative CK19-2G2 level was significantly lower than the preoperative level. An earlier study previously reported that the dynamic changes of serum CK19-2G2 levels before and after chemotherapy were consistent with cancer progression [8]. These results imply that CK19-2G2 might be associated with tumor burden or cell activity. Another study demonstrated that upon release from proliferating or apoptotic cells [9], [10], cytokeratins distinctly reflect ongoing cell activity. Thus, by following patients with repeated assessments of CK19-2G2 levels, in combination with imaging methods or a marker that reflects tumor burden, the oncologist may be able to obtain critical information regarding tumor growth activity. This applies, in particular, to cases where the tumor is already clinically confirmed. In this sense, CK19-2G2 may be a useful marker for monitoring lung cancer patients during treatment and after therapy. However, additional clinical studies are required to fully establish the clinical utility of the CK19-2G2, both alone and in combination with markers that reflect the tumor burden.

In addition, subgroup analyses were performed according to surgery procedures. For patients receiving radical resection, their serum CK19-2G2 level fell from preoperative levels of 10.19 mU/mL to 0.47 mU/mL during the first week after operation. Thereafter, their serum CK19-2G2 concentration declined slightly. However, no statistically significant differences were observed between the T1 and T2 group. From these observations, we deduced that the half-life of CK19-2G2 is relatively short and therefore, its postoperative level could be reduced to a steady, low level within a week after tumor resection. Our deduction is consistent with previous findings. Earlier studies have demonstrated that the half-life of cytokeratin fragments in the circulation is, depending on the size of the fragment, approximately 10–15 h [11], [12]. Therefore, in this study, the assessment of serum CK19-2G2 levels, which was performed a week after tumor resection, could reflect residual tumor activity.

Although no residual neoplasm was observed by imaging examination, the serum CK19-2G2 level of the cancer patients after surgery was still significantly higher than that of healthy controls. This may be due to the micrometastases of tumor cells. Hematogenous metastasis is a main mode of tumor metastasis. Radical tumor resection may have removed the visible tumor, but may have failed to completely eliminate the tumor cells that had disseminated into the peripheral blood. Micrometastases of tumor cells may lead to the tumor recurrence or metastasis. In this sense, CK19-2G2 may be a prognostic marker for lung cancer patients. This deduction has been confirmed in another study on breast cancer. Kong Y *et al.* revealed that serum CK19-2G2 level was an independent factor for relapse and death in primary breast cancer patients, and may be a candidate marker for monitoring metastasis in breast cancer [13]. Similar effects may be present in lung cancer patients.

During the first week after receiving palliative operation, the patients' serum CK19-2G2 levels dropped by almost 80%, compared to the preoperative levels. Thereafter, their serum CK19-2G2 concentrations declined further, and dropped to approximately 10% of their preoperative levels. It was speculated that palliative surgery partly reduced tumor burden, and other factors, such as the postoperative treatments or the body's anti-tumor immunity, could have further eliminated the number and activity of tumor cells. However, the postoperative CK19-2G2 levels in the palliative operation group were significantly higher than that of the radical resection group. The reason may include the following: 1) the patient underwent palliative operation either to treat metastases to other organs or visible residual tumor masses remaining after surgery; 2) postoperative therapy had just been performed when the last blood draw was conducted, and its effect was not yet reflected.

However, there are still several limitations in the present study. First, the ratio of clinical stage was not well balanced. Most cases were in the early stages (I+II: 55.9%), and the percent of IV stage patients was only 2.86%. We compared the CK19-2G2 level by clinical stages, and no significant difference was found (Data not shown). According to these results, we deduced that the disproportional staging of lung cancer patients would not significantly affect the mean level of CK19-2G2. Second, the spectrum of benign pulmonary disease was not broad enough. Due to the professional limitations of a cancer hospital, there are slight differences in disease spectrum observed in general hospitals. Multicenter studies are needed to adjust the possible biases inherent to a single-center study.

In conclusion, by reflecting residual tumor activity, CK19-2G2 is feasible as a tumor marker in diagnosing and monitoring response to treatment in lung cancer patients. In addition, CK19-2G2 may be an indicator for micrometastases in lung cancer patients. However, multicenter studies are needed to verify this conclusion.

## Supporting Information

Table S1 The concentration of CK19-2G2 for lung cancer, benign diseases and healthy controls (mU/mL).
(DOC)

Table S2 Serum CK19-2G2 concentration in the lung cancer group before and after operation (mU/mL).
(DOC)
