# Supplementary material for: Serum Cytokeratin 19 Fragment, CK19-2G2, as a Newly Identified Biomarker for Lung Cancer
Source: PLoS One. 2014 Jul 9;9(7):e101979. doi: 10.1371/journal.pone.0101979 (PMC4090200; doi:10.1371/journal.pone.0101979)
Supplement: Table S1 — The concentration of CK19-2G2 for lung cancer, benign diseases and healthy controls (mU/mL). (DOC) [file pone.0101979.s001.doc]

**Table S1.** The concentration of CK19-2G2 for lung cancer, benign diseases and healthy controls (mU/mL)

| ADC | SCC | SCLC | benign | healthy |
| --- | --- | --- | --- | --- |
| 1.4031 | 12.3382 | 2.7584 | 0.36 | 0.1466 |
| 0.1718 | 1.9352 | 1.6627 | 0.4311 | 0.3155 |
| 0.387 | 1.7303 | 1.0632 | 0.2294 | 0.2791 |
| 7.9395 | 12.171 | 1.8414 | 0.3704 | 0.4325 |
| 2.6414 | 2.6723 | 0.3313 | 0.6208 | 0.2965 |
| 0.3556 | 2.7625 | 0.2099 | 1.2605 | 0.2112 |
| 6.9602 | 1.6318 | 0.8037 | 0.3619 | 0.3691 |
| 1.8456 | 2.3903 | 1.047 | 0.714 | 0.2444 |
| 4.498 | 1.9654 | 0.5431 | 0.4412 | 0.2733 |
| 0.7212 | 11.4702 | 6.61 | 0.1705 | 0.651 |
| 1.8175 | 7.7063 | 0.41 | 0.4018 | 0.2237 |
| 0.2801 | 19.4883 | 0.46 | 0.2486 | 0.4811 |
| 1.8979 | 0.821 | 6.24 | 0.1548 | 0.1579 |
| 0.2001 | 6.2867 | 8.8771 | 0.5463 | 0.3219 |
| 9.6241 | 1.2845 | 10.1525 | 0.4814 | 0.1563 |
| 0.691 | 42.6855 | 5.4389 | 0.3911 | 0.1568 |
| 0.2618 | 3.7381 | 11.5734 | 0.9711 | 0.1024 |
| 1.0478 | 2.0237 | 5.5585 | 0.3417 | 0.2088 |
| 0.9846 | 1.0839 | 11.1419 | 0.5642 | 0.3602 |
| 0.4009 | 18.4059 | 4.747 | 0.415 | 0.3305 |
| 0.7823 | 2.2862 | 6.737 | 0.585 | 0.3992 |
| 0.3524 | 5.5772 | 13.6144 | 0.6621 | 0.17 |
| 4.1717 | 4.2468 | 5.5903 | 0.0667 | 0.2438 |
| 11.5274 | 5.8274 | 22.3115 | 2.9396 | 0.1534 |
| 1.838 | 5.50 | 28.5235 | 0.1455 | 0.3202 |
| 4.4972 | 6.39 | 18.256 | 0.1755 | 0.0547 |
| 1.9771 | 4.08 | 5.0748 | 0.563 | 0.2698 |
| 1.9604 | 7.67 | 4.9796 | 0.6827 | 0.2386 |
| 0.4877 | 4.18 | 4.9639 | 0.1113 | 0.2683 |
| 0.4909 | 14.59 | 4.9397 | 0.359 | 0.4236 |
| 1.6589 | 6.23 | 6.2132 | 0.2675 | 0.1073 |
| 1.865 | 0.9313 | 4.4719 | 0.1946 | 0.3022 |
| 0.382 | 16.0901 | 5.2757 | 0.6416 | 0.2136 |
| 0.5116 | 9.2792 | 3.9742 | 0.4464 | 0.1961 |
| 0.9405 | 10.2888 | 8.0531 | 1.84 | 0.2143 |
| 3.1577 | 4.3493 | 8.3146 | 1.53 | 0.5355 |
| 0.6595 | 6.1453 |  | 0.55 | 0.1288 |
| 0.2234 | 11.3777 |  | 1.96 | 0.2266 |
| 0.3078 | 18.543 |  | 1.87 | 0.5415 |
| 0.5467 | 9.2585 |  | 1.82 | 0.468 |
| 0.669 | 101.5656 |  | 1.88 | 0.6167 |
| 2.2253 | 5.5822 |  | 1.35 | 0.244 |
| 0.5522 | 10.4246 |  | 0.37 | 0.2742 |
| 0.5182 | 127.6252 |  | 0.40 | 0.2058 |
| 1.9967 | 5.8234 |  | 1.7826 | 0.2517 |
| 0.4513 | 17.5851 |  | 1.2087 | 0.2641 |
| 0.8612 | 0.7663 |  | 0.4976 | 0.3031 |
| 5.66 | 7.2864 |  | 0.7674 | 0.3056 |
| 8.13 | 0.7835 |  | 0.3785 | 0.4703 |
| 5.50 | 11.6199 |  | 1.6394 | 0.147 |
| 6.35 | 1.7256 |  | 1.5741 | 0.1087 |
| 4.59 | 3.1967 |  | 1.6463 | 0.4651 |
| 4.53 | 1.6877 |  | 0.5793 | 0.5334 |
| 0.59 | 0.2042 |  | 6.2085 | 0.2966 |
| 5.52 | 4.1392 |  | 1.0331 | 0.1302 |
| 5.90 | 1.0204 |  | 1.6456 | 0.2874 |
| 5.49 | 2.5591 |  | 0.4365 | 0.2109 |
| 3.71 | 0.8151 |  | 1.3421 | 0.1612 |
| 6.59 | 7.7171 |  | 1.1165 | 0.2175 |
| 0.37 | 8.9827 |  | 0.6745 | 0.2818 |
| 3.66 | 1.4283 |  | 1.4432 | 0.3774 |
| 7.87 | 8.8052 |  | 1.2259 | 0.4568 |
| 5.03 | 2.8362 |  | 0.3077 | 0.3521 |
| 7.34 | 6.8466 |  | 0.5295 | 0.2743 |
| 11.51 | 9.1833 |  | 0.8037 | 0.4402 |
| 11.4255 | 6.9092 |  | 1.5865 | 0.1556 |
| 4.59 | 8.4465 |  | 0.6625 | 0.2629 |
| 3.98 | 6.3788 |  | 1.0892 | 0.4169 |
| 6.10 | 7.9362 |  | 0.1413 | 0.2415 |
| 0.64 | 7.6948 |  | 0.1859 | 0.3549 |
| 9.78 | 8.8935 |  | 1.6416 | 0.2611 |
| 11.9972 | 12.6385 |  | 1.9305 | 0.1861 |
| 51.4647 | 6.9974 |  | 1.6907 | 0.0758 |
| 8.1879 | 7.038 |  | 1.148 | 0.1595 |
| 12.2064 | 7.4361 |  | 0.8515 | 0.1374 |
| 9.4526 | 15.7878 |  | 0.892 | 0.2552 |
| 9.3814 | 15.0473 |  | 1.5126 | 0.0955 |
| 11.0943 | 6.843 |  | 1.1247 | 0.1898 |
| 6.6282 | 1.4585 |  | 0.892 | 0.1038 |
| 6.0409 | 4.8175 |  | 0.764 | 0.352 |
| 5.0833 | 8.8162 |  | 1.1575 | 0.0454 |
| 10.891 | 10.2872 |  | 1.0577 | 0.3568 |
| 0.3456 | 2.5545 |  | 0.9159 | 0.1048 |
| 4.2031 | 9.103 |  | 0.7484 | 0.0529 |
| 7.2454 | 6.5346 |  | 0.1696 | 0.0893 |
| 9.2585 | 4.849 |  | 0.0609 | 0.3178 |
| 13.3356 | 4.7158 |  | 0.3971 | 0.0972 |
| 0.2739 | 7.1645 |  | 0.2027 | 0.2121 |
| 6.731 | 44.017 |  | 0.5532 | 0.135 |
| 1.796 | 4.5366 |  | 0.0529 | 0.1089 |
| 11.0193 | 0.8764 |  | 1.9326 | 0.2177 |
| 5.2268 | 7.4062 |  | 3.2845 | 0.4861 |
| 3.6192 | 12.4343 |  | 0.1816 | 0.1855 |
| 9.1153 | 4.6585 |  | 0.2693 | 0.8178 |
| 7.0975 | 6.7496 |  | 0.0385 | 0.3996 |
| 14.1845 | 9.0559 |  | 0.0582 | 0.2478 |
| 1.1951 | 2.0194 |  | 0.2443 | 0.2244 |
| 1.854 | 1.7337 |  | 0.1752 | 0.2928 |
| 9.2644 | 7.332 |  | 0.1197 | 0.2144 |
| 13.7545 | 7.1181 |  | 0.2652 | 0.2274 |
| 6.9787 | 2.6327 |  | 0.3092 | 0.1496 |
| 13.3825 | 1.9101 |  | 0.415 | 0.6495 |
| 1.0675 | 2.243 |  | 0.025 | 0.3692 |
| 1.9905 | 10.2258 |  | 1.0995 | 0.7302 |
| 3.0756 | 3.3227 |  | 0.3257 | 0.1467 |
| 2.7493 | 9.6868 |  | 0.2365 | 0.1017 |
| 11.3298 | 5.272 |  | 0.4606 | 0.1636 |
| 1.8636 | 1.7305 |  | 0.0281 | 0.3199 |
| 6.4406 | 4.2971 |  | 0.1702 | 0.1665 |
| 3.3873 | 17.1354 |  | 0.1494 | 0.4488 |
| 5.331 | 5.1262 |  | 0.2404 | 0.4786 |
| 9.6452 | 3.0458 |  | 0.1001 | 0.5491 |
| 7.3531 | 8.8246 |  | 0.1565 | 0.2027 |
| 5.9668 | 8.1642 |  | 0.0001 | 0.4106 |
| 13.2751 | 18.0263 |  | 0.4205 | 0.3524 |
| 26.8243 | 13.6374 |  | 0.1885 | 0.2823 |
| 8.8795 | 66.8567 |  | 0.0891 | 0.5407 |
| 6.843 | 6.3505 |  | 0.0739 | 0.3224 |
| 7.3569 | 13.5717 |  | 0.106 | 0.4557 |
| 14.0281 | 15.5302 |  | 0.4089 | 0.2992 |
| 12.6124 | 6.6362 |  | 0.1881 | 0.3115 |
| 9.5479 | 7.1365 |  | 0.1669 | 0.2841 |
| 7.5643 | 20.8531 |  | 0.0001 | 0.2462 |
| 10.3997 | 10.3941 |  | 0.0922 | 0.8337 |
| 15.7327 | 6.4189 |  | 0.5433 | 0.2971 |
| 7.4345 | 41.6073 |  | 0.6546 | 0.1681 |
| 5.5235 | 16.1833 |  | 0.757 | 0.3691 |
| 10.3137 | 21.048 |  | 0.1928 | 0.2818 |
| 11.5733 | 31.0213 |  | 0.1744 | 0.1931 |
| 12.2884 | 15.4426 |  | 0.1496 | 0.3812 |
| 8.6697 | 28.8669 |  | 0.2902 | 0.2534 |
| 10.2668 | 41.1278 |  | 0.6285 | 0.3937 |
| 9.5335 | 8.2377 |  | 0.1738 | 0.644 |
| 7.6651 | 48.2141 |  | 0.654 | 0.4088 |
| 8.6475 | 16.7626 |  |  | 0.2067 |
| 10.3677 | 1.1384 |  |  | 0.1875 |
| 7.9422 | 1.4901 |  |  | 0.1479 |
| 10.6127 | 9.7981 |  |  | 0.2626 |
| 7.7221 | 12.5093 |  |  | 0.242 |
| 12.8657 | 15.638 |  |  | 0.5094 |
| 7.4411 | 21.9027 |  |  | 0.2287 |
| 21.7516 | 10.1608 |  |  | 0.3499 |
| 8.7502 | 11.5838 |  |  | 0.1255 |
| 11.6765 | 43.9758 |  |  | 0.2466 |
| 9.2947 | 3.2438 |  |  | 0.2107 |
| 13.6174 | 5.7904 |  |  | 0.9123 |
| 4.8476 | 4.7322 |  |  | 0.489 |
| 12.3106 | 10.5062 |  |  | 1.1616 |
| 11.5245 | 6.4463 |  |  | 0.4441 |
| 8.9114 | 8.1434 |  |  | 0.275 |
| 15.3413 | 14.9089 |  |  | 0.2815 |
| 8.4263 | 12.3729 |  |  | 0.7412 |
| 2.6062 | 47.3323 |  |  | 0.2364 |
| 6.3383 | 12.2847 |  |  | 0.3993 |
| 4.1288 | 17.0388 |  |  | 0.4042 |
| 2.4012 | 15.0836 |  |  | 0.5164 |
| 5.1921 | 12.7229 |  |  | 0.2084 |
| 3.6143 | 4.0302 |  |  | 0.2354 |
| 3.8122 | 10.7386 |  |  | 0.1524 |
| 7.5694 | 10.9421 |  |  | 0.383 |
| 2.0932 | 6.0811 |  |  | 0.3209 |
| 2.3902 | 8.9823 |  |  | 0.4339 |
| 22.5508 | 1.4292 |  |  | 0.2818 |
| 18.7857 | 14.846 |  |  | 0.4577 |
| 5.4735 | 8.0476 |  |  | 0.185 |
| 7.1976 | 1.9858 |  |  | 0.6278 |
| 3.3327 | 9.1455 |  |  | 0.8562 |
| 7.4089 | 6.7548 |  |  | 0.6006 |
| 18.5924 | 4.4012 |  |  | 0.2839 |
| 4.3973 | 36.0405 |  |  | 0.2936 |
| 3.404 | 2.8964 |  |  | 0.3235 |
| 4.9988 | 4.6955 |  |  | 0.2972 |
| 4.0809 | 7.3546 |  |  | 0.126 |
| 6.4422 | 8.3674 |  |  | 0.2781 |
| 7.1623 | 4.8399 |  |  | 0.4569 |
| 4.1575 | 15.2159 |  |  | 0.2838 |
| 5.8284 | 1.9017 |  |  | 0.1131 |
| 7.0261 | 3.828 |  |  | 0.1996 |
| 3.5357 | 28.0765 |  |  | 0.1228 |
| 5.3266 | 0.7255 |  |  | 0.1115 |
| 6.8498 | 17.1957 |  |  | 0.0999 |
| 2.2775 | 19.449 |  |  | 0.3174 |
| 5.4552 | 35.7658 |  |  | 0.1139 |
| 7.043 | 1.1783 |  |  | 0.2249 |
| 3.8281 | 3.7102 |  |  | 0.1013 |
| 2.6102 | 9.5403 |  |  | 0.0111 |
| 9.1435 | 24.7478 |  |  | 0.0171 |
| 3.2994 | 9.0531 |  |  | 0.0917 |
| 3.7981 | 3.5268 |  |  | 0.0701 |
| 5.6847 | 2.0587 |  |  | 0.1333 |
| 6.8033 | 4.1068 |  |  | 0.1923 |
| 3.9213 | 6.23 |  |  | 0.0681 |
| 14.8619 | 2.4503 |  |  | 0.0597 |
| 12.3861 | 3.1613 |  |  | 0.0548 |
| 2.7972 | 7.9362 |  |  | 0.1352 |
| 8.972 | 8.1296 |  |  | 0.162 |
| 3.0759 | 7.5643 |  |  | 0.2175 |
| 10.2362 | 16.8323 |  |  | 0.172 |
| 7.625 | 6.113 |  |  | 0.3162 |
| 23.8094 | 3.444 |  |  | 0.1788 |
| 15.5079 | 15.7327 |  |  | 0.2595 |
| 11.206 | 4.5051 |  |  | 0.2306 |
| 16.52 | 7.9422 |  |  | 0.1816 |
| 4.933 | 7.7221 |  |  | 0.0201 |
| 13.5845 | 15.8126 |  |  | 0.1846 |
| 28.1512 | 21.7516 |  |  | 0.1292 |
| 26.8243 | 2.6083 |  |  | 0.285 |
| 2.7143 | 3.6541 |  |  | 0.051 |
| 10.2411 |  |  |  | 0.116 |
| 13.0266 |  |  |  | 0.0592 |
| 13.6268 |  |  |  | 0.025 |
| 12.8109 |  |  |  | 0.1642 |
| 13.1027 |  |  |  | 0.1981 |
| 28.1692 |  |  |  | 0.1197 |
| 9.0057 |  |  |  | 0.0798 |
| 8.3899 |  |  |  | 0.1087 |
| 19.3004 |  |  |  | 0.0881 |
| 15.6717 |  |  |  | 0.2181 |
| 16.7962 |  |  |  | 0.1558 |
| 9.7956 |  |  |  | 0.3808 |
| 20.48 |  |  |  | 0.2279 |
| 17.6717 |  |  |  | 0.3017 |
| 23.3691 |  |  |  | 0.7714 |
| 23.9646 |  |  |  | 0.1527 |
| 11.734 |  |  |  | 0.2441 |
| 12.3932 |  |  |  | 0.1259 |
| 6.9195 |  |  |  | 0.3204 |
| 6.8937 |  |  |  | 0.0709 |
| 8.0542 |  |  |  | 0.2681 |
| 27.6454 |  |  |  | 0.5428 |
| 32.4283 |  |  |  | 0.1106 |
| 9.2685 |  |  |  | 0.1631 |
| 10.7144 |  |  |  | 0.0166 |
| 16.951 |  |  |  | 0.2048 |
| 9.1626 |  |  |  | 0.0099 |
| 13.3814 |  |  |  | 0.337 |
| 8.2429 |  |  |  | 0.0807 |
| 19.9789 |  |  |  | 0.4082 |
| 26.5582 |  |  |  | 0.0873 |
| 30.6147 |  |  |  | 0.1383 |
| 25.3637 |  |  |  | 0.0523 |
| 14.4488 |  |  |  | 0.2001 |
| 6.9525 |  |  |  | 0.0989 |
| 17.8549 |  |  |  | 0.1676 |
| 23.0853 |  |  |  | 0.0858 |
| 6.8909 |  |  |  | 0.0386 |
| 6.1115 |  |  |  | 1.4015 |
| 9.424 |  |  |  | 0.0539 |
| 5.2038 |  |  |  | 0.1637 |
| 8.3542 |  |  |  | 0.2681 |
| 5.2968 |  |  |  | 0.072 |
| 15.2034 |  |  |  | 0.046 |
| 13.6443 |  |  |  | 0.0748 |
| 23.3558 |  |  |  | 0.1502 |
| 6.8793 |  |  |  | 0.0281 |
| 8.1386 |  |  |  | 0.0212 |
| 16.7858 |  |  |  | 0.1448 |
| 17.3379 |  |  |  | 0.1017 |
| 10.0925 |  |  |  | 0.195 |
| 14.9829 |  |  |  | 0.029 |
| 15.3898 |  |  |  | 0.0699 |
| 16.7369 |  |  |  | 0.0251 |
| 7.0349 |  |  |  | 0.3175 |
| 12.2678 |  |  |  |  |
| 18.6332 |  |  |  |  |
| 22.4183 |  |  |  |  |
| 8.2305 |  |  |  |  |
| 11.2363 |  |  |  |  |
| 14.3975 |  |  |  |  |
| 9.1723 |  |  |  |  |
| 6.1757 |  |  |  |  |
| 12.7989 |  |  |  |  |
| 5.353 |  |  |  |  |
| 8.0689 |  |  |  |  |
| 8.2284 |  |  |  |  |
| 8.2203 |  |  |  |  |
| 15.3223 |  |  |  |  |
| 2.175 |  |  |  |  |
| 12.2302 |  |  |  |  |
| 16.9014 |  |  |  |  |
| 4.7606 |  |  |  |  |
| 1.8463 |  |  |  |  |
| 6.7517 |  |  |  |  |
| 12.7627 |  |  |  |  |
| 2.8807 |  |  |  |  |
| 9.998 |  |  |  |  |
| 6.8982 |  |  |  |  |
| 7.2746 |  |  |  |  |
| 13.4155 |  |  |  |  |
| 8.2259 |  |  |  |  |
| 7.0767 |  |  |  |  |
| 4.1079 |  |  |  |  |
| 13.2371 |  |  |  |  |
| 13.0583 |  |  |  |  |
| 5.5127 |  |  |  |  |
| 10.385 |  |  |  |  |
| 14.7657 |  |  |  |  |
| 13.2108 |  |  |  |  |
| 4.2379 |  |  |  |  |
| 4.414 |  |  |  |  |
| 8.9209 |  |  |  |  |
| 2.1915 |  |  |  |  |
| 18.651 |  |  |  |  |
| 5.4447 |  |  |  |  |
| 11.939 |  |  |  |  |
| 10.7977 |  |  |  |  |
| 2.3697 |  |  |  |  |
| 4.4985 |  |  |  |  |
| 1.1054 |  |  |  |  |
| 2.6913 |  |  |  |  |
| 3.1221 |  |  |  |  |
| 6.9092 |  |  |  |  |
| 3.8591 |  |  |  |  |
| 8.4465 |  |  |  |  |
| 4.7575 |  |  |  |  |
| 7.0249 |  |  |  |  |
| 4.4801 |  |  |  |  |
| 3.6147 |  |  |  |  |
| 2.8554 |  |  |  |  |
| 4.1849 |  |  |  |  |
| 4.7302 |  |  |  |  |
| 3.7636 |  |  |  |  |
| 6.174 |  |  |  |  |
| 5.8375 |  |  |  |  |
| 4.4084 |  |  |  |  |
| 7.4246 |  |  |  |  |
| 4.9607 |  |  |  |  |
| 5.059 |  |  |  |  |
| 0.764 |  |  |  |  |
| 2.5878 |  |  |  |  |
| 4.6615 |  |  |  |  |
| 6.7919 |  |  |  |  |
| 3.8038 |  |  |  |  |
| 1.1546 |  |  |  |  |
| 7.5347 |  |  |  |  |
| 11.4846 |  |  |  |  |
| 4.8759 |  |  |  |  |
| 5.3162 |  |  |  |  |
| 7.3586 |  |  |  |  |
| 5.7489 |  |  |  |  |
| 4.4255 |  |  |  |  |
| 5.6738 |  |  |  |  |
| 6.9887 |  |  |  |  |
| 2.9927 |  |  |  |  |
| 13.6932 |  |  |  |  |
