# Supplementary material for: Serum Cytokeratin 19 Fragment, CK19-2G2, as a Newly Identified Biomarker for Lung Cancer
Source: PLoS One. 2014 Jul 9;9(7):e101979. doi: 10.1371/journal.pone.0101979 (PMC4090200; doi:10.1371/journal.pone.0101979)
Supplement: Table S2 — Serum CK19-2G2 concentration in the lung cancer group before and after operation (mU/mL). (DOC) [file pone.0101979.s002.doc]

**Table S2.** Serum CK19-2G2 concentration in the lung cancer group before and after operation (mU/mL)

| Radical Resection | | | Palliative Operation | | |
| --- | --- | --- | --- | --- | --- |
| Pre-operation (T0) | 1week after operation (T1) | 1 month after operation (T2) | Pre-operation (T0) | 1week after operation (T1) | 1 month after operation (T2) |
| 8.9114 | 0.4028 | 0.1526 | 2.6327 | 1.073 | 0.4115 |
| 2.5545 | 0.2577 | 0.2706 | 9.6868 | 1.9604 | 0.043 |
| 9.103 | 0.2617 | 0.1594 | 2.7972 | 0.9854 | 0.5024 |
| 6.5346 | 0.1003 | 0.1844 | 10.2362 | 0.7545 | 0.1388 |
| 15.3413 | 0.2589 | 0.0705 | 6.4189 | 1.3676 | 4.5783 |
| 4.849 | 0.0552 | 0.1695 | 16.7962 | 0.4469 | 1.4424 |
| 8.4263 | 0.1878 | 0.0946 | 12.3932 | 0.6179 | 0.0001 |
| 2.6062 | 0.1574 | 0.0623 | 8.0542 | 0.5827 | 1.112 |
| 6.3383 | 0.2393 | 0.3568 | 30.6147 | 0.769 | 0.375 |
| 4.1288 | 0.1201 | 0.155 | 43.9758 | 6.4595 | 1.36 |
| 2.4012 | 0.0796 | 0.1278 | 13.6443 | 0.6334 | 0.696 |
| 5.1921 | 0.5028 | 0.0559 | 47.3323 | 33.8077 | 1.265 |
| 3.6143 | 0.2286 | 0.2602 | 6.8793 | 0.3371 |  |
| 3.8122 | 0.1715 | 0.3312 | 12.2847 | 0.3468 |  |
| 7.5694 | 0.0025 | 0.6191 | 10.9421 | 0.3925 |  |
| 2.2298 | 0.0256 | 0.1666 | 9.1723 | 0.533 |  |
| 4.7158 | 0.1019 | 0.3027 | 6.1757 | 0.259 |  |
| 2.0932 | 0.1835 | 0.1058 | 12.7989 | 0.2672 |  |
| 2.3902 | 2.1797 | 0.105 | 14.846 | 9.1203 |  |
| 22.5508 | 0.206 | 0.1359 | 4.9639 | 0.4423 |  |
| 7.1645 | 0.0275 | 0.1549 | 13.0583 | 0.1387 |  |
| 18.7857 | 0.2553 | 0.071 | 1.1783 | 0.9368 |  |
| 44.017 | 0.1443 | 0.217 | 4.414 | 0.0692 |  |
| 5.4735 | 0.0907 | 0.2711 | 2.8554 | 2.6094 |  |
| 7.1976 | 0.1689 | 0.4716 | 3.444 | 0.6733 |  |
| 4.5366 | 0.3903 | 1.2777 | 3.7636 | 0.171 |  |
| 3.3327 | 0.0806 | 0.1322 | 4.9607 | 0.636 |  |
| 7.4089 | 0.3381 | 0.0708 | 6.7919 | 0.351 |  |
| 18.5924 | 0.8989 | 0.0251 | 1.1546 | 0.656 |  |
| 4.3973 | 0.0001 | 0.0922 | 4.4255 | 0.473 |  |
| 0.8764 | 0.8776 | 0.4363 | 2.9927 | 0.571 |  |
| 5.6198 | 0.6241 | 0.1663 |  |  |  |
| 3.404 | 2.6589 | 0.2696 |  |  |  |
| 4.9988 | 0.2998 | 1.618 |  |  |  |
| 4.0809 | 0.1057 | 0.1666 |  |  |  |
| 6.4422 | 0.2231 | 0.2624 |  |  |  |
| 4.6348 | 0.0844 | 0.6647 |  |  |  |
| 7.4062 | 1.0504 | 0.3674 |  |  |  |
| 7.1623 | 0.4746 | 3.1626 |  |  |  |
| 4.1575 | 0.5621 | 0.1655 |  |  |  |
| 12.4343 | 0.11 | 0.3258 |  |  |  |
| 4.6585 | 0.0763 | 0.1917 |  |  |  |
| 6.7496 | 0.3027 | 1.713 |  |  |  |
| 5.8284 | 0.1278 | 0.3311 |  |  |  |
| 9.0559 | 0.0691 | 0.5452 |  |  |  |
| 2.0194 | 0.3048 | 0.0252 |  |  |  |
| 7.0261 | 0.1959 | 0.4301 |  |  |  |
| 3.5357 | 0.098 | 0.2441 |  |  |  |
| 5.3266 | 0.4519 | 0.1855 |  |  |  |
| 6.8498 | 0.0436 | 1.0384 |  |  |  |
| 2.2775 | 0.5732 | 0.4745 |  |  |  |
| 5.4552 | 0.0001 | 0.2859 |  |  |  |
| 1.7337 | 0.2054 | 0.7248 |  |  |  |
| 7.043 | 0.1706 | 0.0887 |  |  |  |
| 3.8281 | 0.2645 | 0.2121 |  |  |  |
| 2.6102 | 0.4418 | 0.301 |  |  |  |
| 7.332 | 0.3252 | 0.1967 |  |  |  |
| 9.1435 | 0.1168 | 0.6401 |  |  |  |
| 3.2994 | 0.3335 | 0.4422 |  |  |  |
| 7.1181 | 0.7835 | 0.8618 |  |  |  |
| 3.7981 | 0.1392 | 0.7349 |  |  |  |
| 5.6847 | 0.1629 | 0.0303 |  |  |  |
| 6.8033 | 0.2991 | 0.401 |  |  |  |
| 3.9213 | 0.5975 | 0.448 |  |  |  |
| 6.1589 | 0.6274 | 0.35 |  |  |  |
| 1.9101 | 0.1148 | 0.502 |  |  |  |
| 2.243 | 0.0895 | 0.744 |  |  |  |
| 10.2258 | 0.0188 | 0.446 |  |  |  |
| 3.3227 | 0.5062 | 0.392 |  |  |  |
| 13.6144 | 0.3495 | 0.573 |  |  |  |
| 14.8619 | 0.3061 | 0.707 |  |  |  |
| 12.3861 | 0.7336 | 0.842 |  |  |  |
| 5.272 | 0.0001 | 0.656 |  |  |  |
| 5.5903 | 0.2107 | 0.0001 |  |  |  |
| 8.972 | 0.4839 | 0.862 |  |  |  |
| 3.0759 | 0.1879 | 0.334 |  |  |  |
| 4.1026 | 0.2526 | 0.386 |  |  |  |
| 7.625 | 0.3004 | 0.476 |  |  |  |
| 1.7305 | 0.3176 | 0.672 |  |  |  |
| 23.8094 | 0.3721 | 0.616 |  |  |  |
| 4.2971 | 0.1925 | 0.488 |  |  |  |
| 15.5079 | 0.6184 | 0.4 |  |  |  |
| 17.1354 | 0.3698 | 0.428 |  |  |  |
| 11.206 | 0.4911 | 0.674 |  |  |  |
| 5.1262 | 4.0276 | 0.669 |  |  |  |
| 16.52 | 0.3199 | 0.506 |  |  |  |
| 3.0458 | 0.3719 | 0.488 |  |  |  |
| 8.8246 | 0.1668 | 0.451 |  |  |  |
| 4.933 | 0.5449 | 0.448 |  |  |  |
| 8.1642 | 0.2504 |  |  |  |  |
| 18.0263 | 1.0922 |  |  |  |  |
| 13.5845 | 0.2063 |  |  |  |  |
| 13.6374 | 0.8309 |  |  |  |  |
| 28.1512 | 0.3136 |  |  |  |  |
| 26.8243 | 1.0172 |  |  |  |  |
| 2.7143 | 0.0539 |  |  |  |  |
| 66.8567 | 0.8181 |  |  |  |  |
| 6.3505 | 0.4682 |  |  |  |  |
| 10.2411 | 0.3947 |  |  |  |  |
| 13.5717 | 0.2904 |  |  |  |  |
| 13.0266 | 0.2062 |  |  |  |  |
| 13.6268 | 0.7696 |  |  |  |  |
| 15.5302 | 0.2587 |  |  |  |  |
| 12.8109 | 0.3901 |  |  |  |  |
| 6.6362 | 0.2086 |  |  |  |  |
| 7.1365 | 0.3653 |  |  |  |  |
| 13.1027 | 0.6898 |  |  |  |  |
| 20.8531 | 0.2233 |  |  |  |  |
| 28.1692 | 0.2957 |  |  |  |  |
| 6.8331 | 0.7618 |  |  |  |  |
| 9.0057 | 0.497 |  |  |  |  |
| 8.3899 | 0.3797 |  |  |  |  |
| 10.3941 | 0.8667 |  |  |  |  |
| 41.6073 | 0.1832 |  |  |  |  |
| 16.1833 | 6.3405 |  |  |  |  |
| 19.3004 | 0.3775 |  |  |  |  |
| 21.048 | 0.2897 |  |  |  |  |
| 31.0213 | 0.3283 |  |  |  |  |
| 15.6717 | 0.2111 |  |  |  |  |
| 15.4426 | 0.1693 |  |  |  |  |
| 9.7956 | 0.1518 |  |  |  |  |
| 28.8669 | 0.3476 |  |  |  |  |
| 20.48 | 0.5433 |  |  |  |  |
| 41.1278 | 1.4863 |  |  |  |  |
| 8.2377 | 0.5971 |  |  |  |  |
| 17.6717 | 0.2161 |  |  |  |  |
| 23.3691 | 0.1263 |  |  |  |  |
| 23.9646 | 0.3065 |  |  |  |  |
| 11.734 | 0.4239 |  |  |  |  |
| 48.2141 | 0.3122 |  |  |  |  |
| 6.9195 | 0.1507 |  |  |  |  |
| 6.8937 | 0.2773 |  |  |  |  |
| 16.7626 | 0.2984 |  |  |  |  |
| 1.1384 | 0.5969 |  |  |  |  |
| 27.6454 | 0.2605 |  |  |  |  |
| 1.4901 | 0.7249 |  |  |  |  |
| 9.7981 | 0.5092 |  |  |  |  |
| 32.4283 | 0.1619 |  |  |  |  |
| 9.2685 | 0.1173 |  |  |  |  |
| 22.3115 | 1.828 |  |  |  |  |
| 10.7144 | 0.1665 |  |  |  |  |
| 12.5093 | 0.2236 |  |  |  |  |
| 16.951 | 0.7703 |  |  |  |  |
| 28.5235 | 0.7128 |  |  |  |  |
| 9.1626 | 0.2453 |  |  |  |  |
| 19.2731 | 0.3177 |  |  |  |  |
| 13.3814 | 0.2462 |  |  |  |  |
| 15.0185 | 0.18 |  |  |  |  |
| 8.2429 | 0.5686 |  |  |  |  |
| 19.9789 | 0.8478 |  |  |  |  |
| 15.638 | 0.3323 |  |  |  |  |
| 21.9027 | 0.3866 |  |  |  |  |
| 13.5353 | 0.2807 |  |  |  |  |
| 16.0592 | 0.322 |  |  |  |  |
| 26.5582 | 0.22 |  |  |  |  |
| 10.1608 | 0.3541 |  |  |  |  |
| 11.5838 | 0.542 |  |  |  |  |
| 25.3637 | 0.3286 |  |  |  |  |
| 14.4488 | 0.359 |  |  |  |  |
| 3.2438 | 0.6979 |  |  |  |  |
| 6.9525 | 0.2788 |  |  |  |  |
| 17.8549 | 0.399 |  |  |  |  |
| 5.7904 | 1.2458 |  |  |  |  |
| 23.0853 | 0.1748 |  |  |  |  |
| 6.8909 | 0.6519 |  |  |  |  |
| 4.7322 | 0.2851 |  |  |  |  |
| 10.5062 | 0.5931 |  |  |  |  |
| 6.1115 | 0.4943 |  |  |  |  |
| 3.7671 | 0.466 |  |  |  |  |
| 6.4463 | 0.1897 |  |  |  |  |
| 8.1434 | 1.1148 |  |  |  |  |
| 14.9089 | 0.4066 |  |  |  |  |
| 9.424 | 0.3457 |  |  |  |  |
| 5.2038 | 0.2542 |  |  |  |  |
| 8.3542 | 0.2292 |  |  |  |  |
| 5.2968 | 0.654 |  |  |  |  |
| 12.3729 | 0.2021 |  |  |  |  |
| 15.2034 | 0.2252 |  |  |  |  |
| 18.256 | 0.2391 |  |  |  |  |
| 23.3558 | 0.3946 |  |  |  |  |
| 17.0388 | 0.2965 |  |  |  |  |
| 8.1386 | 0.4805 |  |  |  |  |
| 15.0836 | 0.423 |  |  |  |  |
| 12.7229 | 0.3355 |  |  |  |  |
| 16.7858 | 0.7269 |  |  |  |  |
| 17.3379 | 0.5788 |  |  |  |  |
| 8.1816 | 0.2694 |  |  |  |  |
| 10.0925 | 0.2289 |  |  |  |  |
| 14.9829 | 0.3486 |  |  |  |  |
| 15.3898 | 0.2138 |  |  |  |  |
| 16.7369 | 0.1058 |  |  |  |  |
| 7.0349 | 0.4648 |  |  |  |  |
| 4.0302 | 0.408 |  |  |  |  |
| 12.2678 | 0.3819 |  |  |  |  |
| 18.6332 | 0.2996 |  |  |  |  |
| 22.4183 | 1.7406 |  |  |  |  |
| 8.2305 | 0.9199 |  |  |  |  |
| 10.7386 | 0.3321 |  |  |  |  |
| 6.0811 | 0.0729 |  |  |  |  |
| 11.2363 | 0.2968 |  |  |  |  |
| 14.3975 | 0.7359 |  |  |  |  |
| 8.9823 | 0.5445 |  |  |  |  |
| 5.353 | 0.5206 |  |  |  |  |
| 5.0748 | 1.0732 |  |  |  |  |
| 8.0689 | 1.6685 |  |  |  |  |
| 8.2284 | 0.4895 |  |  |  |  |
| 1.4292 | 0.2858 |  |  |  |  |
| 2.6549 | 0.0661 |  |  |  |  |
| 8.0476 | 0.3736 |  |  |  |  |
| 1.9858 | 0.9406 |  |  |  |  |
| 8.2203 | 0.708 |  |  |  |  |
| 9.1455 | 1.0123 |  |  |  |  |
| 15.3223 | 0.0505 |  |  |  |  |
| 2.175 | 0.2208 |  |  |  |  |
| 6.7548 | 0.1427 |  |  |  |  |
| 4.4012 | 0.3007 |  |  |  |  |
| 12.2302 | 0.4058 |  |  |  |  |
| 4.9796 | 0.1615 |  |  |  |  |
| 16.9014 | 0.343 |  |  |  |  |
| 36.0405 | 1.2947 |  |  |  |  |
| 4.7606 | 0.3636 |  |  |  |  |
| 1.8463 | 0.1535 |  |  |  |  |
| 6.7517 | 0.6627 |  |  |  |  |
| 2.8964 | 0.3826 |  |  |  |  |
| 12.7627 | 0.3749 |  |  |  |  |
| 2.8807 | 0.5427 |  |  |  |  |
| 9.998 | 0.7257 |  |  |  |  |
| 4.6955 | 0.2549 |  |  |  |  |
| 7.3546 | 0.4442 |  |  |  |  |
| 6.8982 | 0.154 |  |  |  |  |
| 7.2746 | 0.0613 |  |  |  |  |
| 8.3674 | 1.3248 |  |  |  |  |
| 4.8399 | 0.1078 |  |  |  |  |
| 13.4155 | 0.3743 |  |  |  |  |
| 15.2159 | 0.0425 |  |  |  |  |
| 8.2259 | 0.4492 |  |  |  |  |
| 7.0767 | 0.17 |  |  |  |  |
| 1.9017 | 0.1845 |  |  |  |  |
| 3.828 | 0.2724 |  |  |  |  |
| 4.1079 | 0.1335 |  |  |  |  |
| 13.2371 | 0.3357 |  |  |  |  |
| 5.5127 | 0.2583 |  |  |  |  |
| 28.0765 | 0.1045 |  |  |  |  |
| 4.9397 | 0.0532 |  |  |  |  |
| 0.7255 | 0.2313 |  |  |  |  |
| 10.385 | 0.2578 |  |  |  |  |
| 17.1957 | 0.3156 |  |  |  |  |
| 19.449 | 0.3947 |  |  |  |  |
| 35.7658 | 0.2175 |  |  |  |  |
| 14.7657 | 0.3857 |  |  |  |  |
| 3.7102 | 0.2243 |  |  |  |  |
| 13.2108 | 0.1 |  |  |  |  |
| 4.2379 | 0.2478 |  |  |  |  |
| 8.9209 | 0.2714 |  |  |  |  |
| 9.5403 | 0.2758 |  |  |  |  |
| 24.7478 | 0.5461 |  |  |  |  |
| 9.0531 | 0.4524 |  |  |  |  |
| 2.1915 | 0.2259 |  |  |  |  |
| 18.651 | 0.1914 |  |  |  |  |
| 5.4447 | 0.1923 |  |  |  |  |
| 11.939 | 0.2209 |  |  |  |  |
| 10.7977 | 0.2719 |  |  |  |  |
| 2.3697 | 0.349 |  |  |  |  |
| 3.5268 | 0.1672 |  |  |  |  |
| 2.0587 | 0.724 |  |  |  |  |
| 4.4985 | 1.6341 |  |  |  |  |
| 1.1054 | 0.4914 |  |  |  |  |
| 2.6913 | 0.0001 |  |  |  |  |
| 3.1221 | 3.5071 |  |  |  |  |
| 6.9092 | 0.5927 |  |  |  |  |
| 6.2132 | 0.9913 |  |  |  |  |
| 4.1068 | 0.6196 |  |  |  |  |
| 3.8591 | 0.4325 |  |  |  |  |
| 6.23 | 0.5855 |  |  |  |  |
| 8.4465 | 1.0308 |  |  |  |  |
| 4.7575 | 0.1347 |  |  |  |  |
| 7.0249 | 0.6267 |  |  |  |  |
| 4.4801 | 0.4557 |  |  |  |  |
| 2.4503 | 0.4225 |  |  |  |  |
| 3.6147 | 0.4671 |  |  |  |  |
| 3.1613 | 0.3222 |  |  |  |  |
| 7.9362 | 0.7302 |  |  |  |  |
| 3.874 | 0.8243 |  |  |  |  |
| 4.4719 | 0.342 |  |  |  |  |
| 5.2757 | 0.3309 |  |  |  |  |
| 8.1296 | 0.2615 |  |  |  |  |
| 7.5643 | 0.6065 |  |  |  |  |
| 16.8323 | 1.0667 |  |  |  |  |
| 6.113 | 0.5362 |  |  |  |  |
| 4.1849 | 0.3663 |  |  |  |  |
| 4.7302 | 0.3391 |  |  |  |  |
| 5.1101 | 0.7155 |  |  |  |  |
| 15.7327 | 1.188 |  |  |  |  |
| 4.5051 | 0.3055 |  |  |  |  |
| 3.9742 | 0.392 |  |  |  |  |
| 6.174 | 0.632 |  |  |  |  |
| 5.8375 | 0.538 |  |  |  |  |
| 4.4084 | 0.644 |  |  |  |  |
| 7.4246 | 0.517 |  |  |  |  |
| 8.0531 | 0.52 |  |  |  |  |
| 5.059 | 0.396 |  |  |  |  |
| 0.764 | 0.569 |  |  |  |  |
| 2.5878 | 0.897 |  |  |  |  |
| 4.6615 | 0.0001 |  |  |  |  |
| 8.3146 | 0.531 |  |  |  |  |
| 3.8038 | 0.0001 |  |  |  |  |
| 7.9422 | 0.38 |  |  |  |  |
| 7.5347 | 0.619 |  |  |  |  |
| 7.7221 | 0.709 |  |  |  |  |
| 15.8126 | 0.316 |  |  |  |  |
| 11.4846 | 1.819 |  |  |  |  |
| 4.8759 | 0.934 |  |  |  |  |
| 5.3162 | 0.525 |  |  |  |  |
| 21.7516 | 0.606 |  |  |  |  |
| 7.3586 | 0.691 |  |  |  |  |
| 5.7489 | 0.0001 |  |  |  |  |
| 2.6083 | 1.112 |  |  |  |  |
| 3.6541 | 1.639 |  |  |  |  |
| 5.6738 | 0.535 |  |  |  |  |
| 6.9887 | 0.515 |  |  |  |  |
| 13.6932 | 0.357 |  |  |  |  |
